# Neuroinflammation: Mechanisms, Dual Roles, and Therapeutic Strategies in Neurological Disorders

**DOI:** 10.3390/cimb47060417

**Published:** 2025-06-04

**Authors:** Mario García-Domínguez

**Affiliations:** 1 Program of Immunology and Immunotherapy, CIMA-Universidad de Navarra, 31008 Pamplona, Spain; mgdom@unav.es; 2 Department of Immunology and Immunotherapy, Clínica Universidad de Navarra, 31008 Pamplona, Spain; 3 Centro de Investigación Biomédica en Red de Cáncer (CIBERONC), 28029 Madrid, Spain

**Keywords:** neuroinflammation, central nervous system, glial cells, blood–brain barrier, neurodegenerative diseases

## Abstract

Neuroinflammation represents a fundamental component in the development and progression of a wide range of neurological disorders, including neurodegenerative diseases, psychiatric conditions, and cerebral injuries. This review examines the complex mechanisms underlying neuroinflammatory responses, with a focus on the interactions between glial cells and neurons. The dualistic role of neuroinflammation is further investigated, highlighting its ability to promote neuroprotection in acute phases while also contributing to neuronal injury and degeneration during chronic activation. This review also considers innovative therapeutic approaches designed to target neuroinflammatory processes, like drug-based treatments and immune-modulating therapies. A thorough understanding of the regulatory balance within neuroinflammatory networks is essential for the development of effective treatments for several neurological pathologies. Finally, this review provides an integrative summary of current evidence and highlights emerging directions in neuroinflammation research.

## 1. Introduction

Neuroinflammation is a multifaceted biological response within the central nervous system (CNS) that has gained recognition as a pivotal contributor to the pathogenesis of numerous neurological diseases [1]. Once mainly seen as a transient protective response to infections, trauma, or neurotoxic injury, neuroinflammation is now understood to have a dual function (either maintaining CNS homeostasis or, on the other hand, driving neurodegeneration) [2,3]. This functional dichotomy is driven by a complex interaction between cellular components and molecular signaling pathways operating within the immune-privileged environment of the CNS [4,5].

At the cellular level, glial cells, particularly microglia and astrocytes, are essential in the regulation of the neuroinflammatory response [6]. Microglia, the resident innate immune cells of the CNS, typically sustain a surveillance phenotype under homeostatic conditions, constantly monitoring the neural milieu [7]. Upon exposure to pathological stimuli, like pathogen-associated molecular patterns (PAMPs) or damage-associated molecular patterns (DAMPs) [8,9], microglia undergo a pronounced phenotypic shift, characterized by the release of pro-inflammatory mediators (e.g., TNF-α, IL-1β, and IL-6), reactive oxygen species (ROS), and nitric oxide (NO) [10,11,12]. These responses are regulated by the activation of some intracellular signaling pathways, including NF-κB, MAPKs (ERK, JNK, and p38), and the formation of the NLRP3 inflammasome [13,14,15]. Pattern recognition receptors (PRRs), such as Toll-like receptors (TLRs) and NOD-like receptors (NLRs), act as critical upstream sensors that trigger these cascades [16,17]. Astrocytes, once considered passive support cells, are now recognized as active contributors to the modulation of CNS inflammation [18]. Astrocytes, driven by microglial-derived factors such as IL-1α, TNF-α, and C1q, lose neuroprotective properties and secrete neurotoxic factors that compromise oligodendrocyte and neuronal viability [19,20]. Additionally, astrocytes regulate the permeability of the blood–brain barrier (BBB) through the production and secretion of VEGF and MMPs, thereby allowing the infiltration of peripheral immune cells, including T lymphocytes and monocytes [21]. On the other hand, neurons are not simply passive recipients of inflammatory signals but actively influence and regulate neuroimmune dynamics. This cell type expresses molecules such as CD200, CX3CL1, and neuregulins, which interact with glial receptors to maintain microglia in a homeostatic state [22,23,24]. However, persistent inflammatory stimuli suppress these regulatory signals, favoring a pro-inflammatory CNS environment [25].

At the molecular level, neuroinflammatory signaling involves a complex network of several transcription factors (such as NF-κB, STATs, and IRFs), kinases, and lipid mediators [26,27,28]. Bioactive lipids such as prostaglandins (notably PGE2) and leukotrienes (synthesized via COX and LOX pathways) amplify inflammatory responses [29,30]. Furthermore, alterations in kynurenine metabolism, a major pathway of tryptophan catabolism, lead to the accumulation of neurotoxic metabolites such as quinolinic acid, which exacerbate excitotoxicity through NMDA receptor overactivation [31].

Chronic neuroinflammation, in contrast with its acute counterpart, is now recognized as a central pathological mechanism in numerous neurodegenerative diseases [32]. In Alzheimer’s disease (AD), maintained microglial activation contributes to amyloid-beta (Aβ) plaques and τ-hyperphosphorylation [33]. In Parkinson’s disease (PD), inflammatory processes exacerbate dopaminergic neuronal loss in the substantia nigra, accompanied by elevated cytokine levels and oxidative stress [34]. In the same way, in traumatic brain injury (TBI) and ischemic stroke, the release of DAMPs initiates prolonged glial activation, secondary neurotoxicity, and lasting cognitive deficits [35,36]. Moreover, neuroinflammation is increasingly recognized as a critical contributor to the pathophysiology of several pediatric neurological disorders, including neuronal ceroid lipofuscinoses (NCLs) and Lafora disease (LD) [37,38]. In these conditions, persistent activation of immune pathways within the CNS underlies progressive neurodegeneration, resulting in the progressive worsening of cognitive and motor impairments. Emerging data indicate that the sustained presence of pro-inflammatory mediators not only reflects neuronal injury but may also play a causative role in driving disease progression during neurodevelopment [39]. Emerging evidence also implicates systemic factors (including gut–brain axis dysfunction and peripheral immune activation) as significant modulators of CNS inflammation [40,41].

Despite the complexity of neuroimmune signaling, several therapeutic strategies are under investigation. These include NLRP3 inhibitors (e.g., MCC950), tetracycline derivatives (e.g., minocycline), glucocorticoids, and biologics targeting key cytokines such as IL-1β (e.g., anakinra) and TNF-α (e.g., etanercept) [42,43,44,45]. Moreover, novel therapeutic strategies are focused on enhancing the resolution phase of inflammation through specialized pro-resolving mediators (SPMs), including resolvins, protectins, and maresins, which promote tissue repair while minimizing immunosuppression [46,47]. A failure in these resolution mechanisms can perpetuate chronic inflammation, cellular stress, and ongoing neurodegeneration [48].

In this context, the present review aims to provide an in-depth analysis of the cellular and molecular mechanisms governing neuroinflammation, emphasizing its dual role in maintaining CNS homeostasis and promoting neurodegeneration. Moreover, this review will explore its involvement in some neurological disorders, as well as current and emerging therapeutic strategies targeting inflammatory pathways. Gaining an understanding of these mechanisms is crucial for the development of more effective treatments for neurological diseases linked to neuroinflammation. Such insights may also contribute to early diagnosis and prevention strategies, ultimately improving patient outcomes.

## 2. Mechanisms of Neuroinflammation

Neuroinflammation represents a highly regulated and complex response of the CNS to diverse stimuli like infections, injuries, and neurodegenerative events [1]. While acute, well-regulated inflammation can support tissue repair and homeostasis, dysregulated inflammatory activity is a hallmark of various CNS pathologies, including AD and PD [2,3,4,5,6,7,8,9,10,11,12,13,14,15,16,17,18,19,20,21,22,23,24,25,26,27,28,29,30,31,32,33,34]. The orchestration of this biological response relies on the dynamic crosstalk among glial cells within the CNS, peripheral immune components, soluble pro-inflammatory factors, and complex intracellular signaling cascades. These pathways initiate and sustain the inflammatory response, ultimately influencing neuronal viability and function.

### 2.1. Cellular Mediators

**Figure 1:**
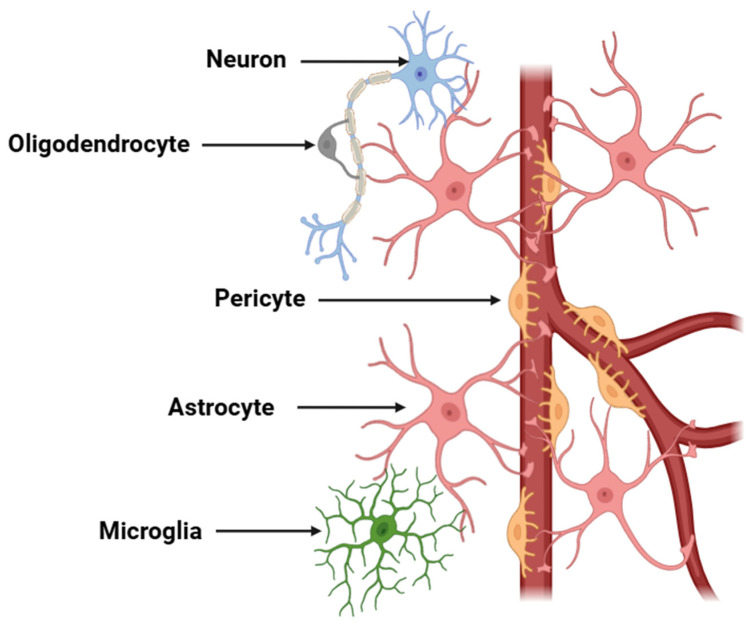
Schematic representation of a neurovascular unit. The diagram illustrates the components of a neurovascular unit, which include neurons, astrocytes, endothelial cells, pericytes, microglia, and the basement membrane. This unit is critical for maintaining cerebral homeostasis, regulating BBB permeability, and facilitating neurovascular coupling. Neuronal activity is coupled with vascular responses via astrocytic end-feet and endothelial signaling mechanisms.

Neurons, the fundamental building blocks of the nervous system, are highly specialized cells responsible for transmitting electrical and chemical signals throughout the body [49]. These specialized cells establish highly complex neural networks that mediate signal transmission between distinct regions of the brain and spinal cord, thereby orchestrating the full spectrum of cognitive, sensory, and motor functions [50]. Neurons are characterized by their unique structure, which includes a cell body, dendrites that receive signals, and an axon that transmits signals to other neurons or muscles [51]. However, these cells do not operate in isolation but interact with a variety of other cells, including glial cells (microglia, astroglia, and oligodendroglia), which provide structural support, protection, and nourishment (Figure 1) [52].

Microglial cells are the resident immune cells of the CNS, playing a key role in maintaining homeostasis, modulating neuronal function, and responding to pathological conditions [53]. These glial cells are derived from yolk sac progenitors early in development and persist throughout life, acting as the first line of defense against injury or infection in the brain and spinal cord [54]. Under physiological conditions, microglial cells maintain a surveillant state, extending and retracting their processes to continuously survey the surrounding microenvironment for signs of homeostatic imbalance [55]. In response to various signals, including injury, infection, or neurodegeneration, microglia can become activated, transforming into a more amoeboid morphology [56]. This activation is characterized by the release of many pro-inflammatory mediators, which can lead to a cascade of neuroinflammatory responses [57]. While microglial activation is fundamental for initiating the inflammatory response to protect the CNS from harmful stimuli, chronic activation of microglia is detrimental [58]. Sustained neuroinflammation, driven by overactive microglia, contributes to neuronal damage, synaptic loss, and even cell death, perpetuating a cycle of chronic inflammation that exacerbates neurodegenerative processes. Under neuroinflammatory conditions, microglia can adopt different polarization states (pro-inflammatory -M1- or anti-inflammatory -M2-) depending on the pathological context and the specific signals present in the microenvironment [59]. The balance between these phenotypes is crucial in determining the trajectory of the inflammatory response: an M1-dominant state may exacerbate neurodegeneration, whereas an M2-dominant profile is associated with tissue repair and neuroprotective effects [60,61]. Moreover, the activation of microglia in neuroinflammation has been associated with changes in BBB permeability, increased oxidative stress, and the release of neurotoxic factors, all of which contribute to a disrupted neuronal environment [62].

Astrocytes, a major class of glial cells, play essential roles in maintaining CNS homeostasis, providing metabolic and structural support to neurons, and modulating synaptic transmission and plasticity [63]. These stellate cells are widely distributed throughout the brain and spinal cord, where they execute numerous vital functions, such as regulating the integrity of the BBB, maintaining ionic equilibrium, clearing excess neurotransmitters, and supporting cellular energy metabolism [64]. Astrocytes are essential for maintaining the extracellular environment, offering structural support to neurons, and serving as intermediaries in the communication between neurons and blood vessels [65]. During neuroinflammation, astrocytes may undergo “reactive astrogliosis”, a process in which they alter their morphology, gene expression, and function in response to various pathological stimuli [66]. While reactive astrocytes attempt to protect the CNS by secreting pro-inflammatory molecules and promoting tissue repair, this response can also have significant deleterious effects when it becomes chronic [67]. In neuroinflammatory states, astrocytes release many pro-inflammatory molecules, which can exacerbate neuronal damage, synaptic dysfunction, and BBB disruption [68,69]. Moreover, reactive astrocytes can engage in bidirectional interactions with microglia, thereby amplifying the inflammatory response and contributing to a self-perpetuating cycle of neural damage and repair that ultimately compromises neuroprotective mechanisms [70]. The balance between neuroinflammation and neuroprotection in astrocytes is delicate, as excessive or prolonged activation of these cells can lead to neuronal death, glial scar formation, and impaired recovery [71].

Finally, oligodendrocytes are a specialized class of glial cells within the CNS, chiefly responsible for the formation of myelin sheaths that insulate axons and ensure the propagation of the electrical signals [72]. Oligodendrocytes originate from oligodendrocyte precursor cells (OPCs), which are distributed throughout the CNS and have the capacity to differentiate into mature oligodendrocytes in response to some cues [73]. The myelination process is essential for the proper functioning of the nervous system, and any disruption in the production or maintenance of myelin can lead to severe neurological conditions, including multiple sclerosis (MS), leukodystrophies, and other demyelinating diseases [74,75]. In situations of neuroinflammation, oligodendrocytes become critically affected. During the neuroinflammation process, pro-inflammatory cytokines exert cytotoxic effects on oligodendrocytes, leading to impaired myelin regeneration and promoting neurodegenerative processes [76]. These molecules can also alter the delicate balance between oligodendrocyte progenitor cell differentiation and oligodendrocyte maturation, inhibiting the remyelination process that is crucial for recovery following demyelination [77]. In diseases such as MS, the immune system erroneously attacks the myelin-producing oligodendrocytes, leading to widespread inflammation that disrupts the normal function of oligodendrocytes [78]. In this context, oligodendrocytes not only become the target of immune-mediated destruction but also play an active role in modulating inflammation. They can secrete various signaling molecules that either exacerbate or resolve the inflammatory response [79]. For instance, oligodendrocytes may release factors that promote the activation of microglia, which can further worsen inflammation [80]. However, oligodendrocytes can also secrete several anti-inflammatory molecules that regulate microglial homeostasis [81].

### 2.2. Pro-Inflammatory Mediators and Their Signaling Pathways

**Figure 2:**
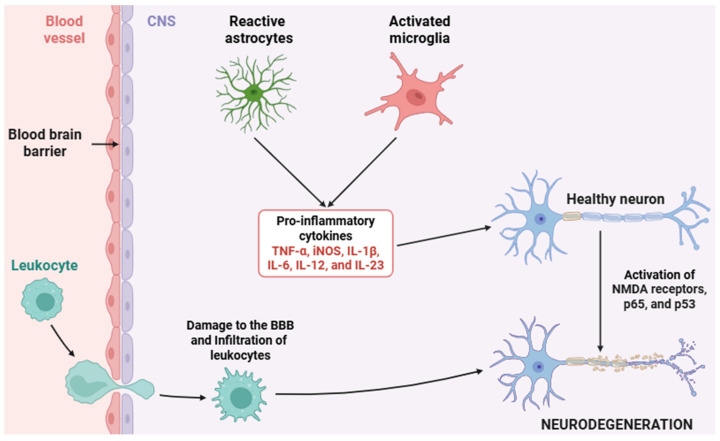
Schematic representation of the neuroinflammatory process. This diagram depicts the key cellular and molecular events involved in neuroinflammation, including the activation of microglia and astrocytes, the release of several pro-inflammatory cytokines, and the recruitment of peripheral immune cells. These processes contribute to BBB disruption, neuronal dysfunction, and tissue damage. Abbreviations: CNS (central nervous system), BBB (blood–brain barrier), TNF-α (tumor necrosis factor alpha), iNOS (inducible nitric oxide synthase), IL-1β (interleukin 1 beta), IL-6 (interleukin 6), IL-12 (interleukin 12), IL-23 (interleukin 23), NMDA (N-methyl-D-aspartate), p65 (NF-κB p65 -RelA- subunit), and p53 (tumor protein 53).

**Figure 3:**
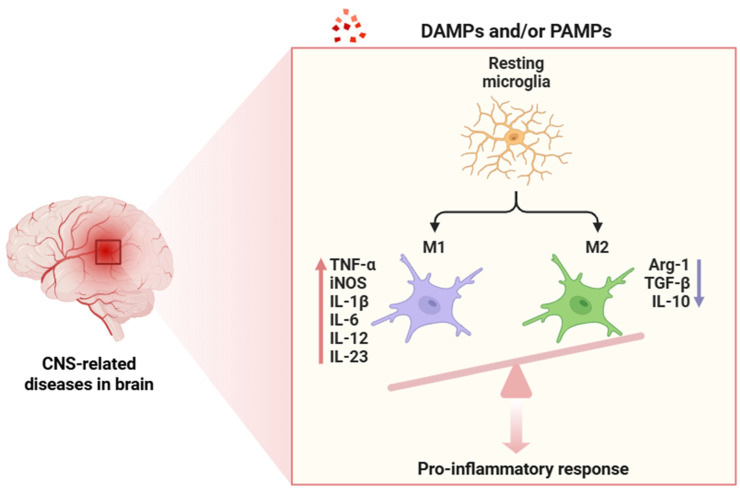
Activation process of resident microglia following exposure to DAMPs or PAMPs. This interaction triggers a strong polarization toward the pro-inflammatory M1 phenotype, whereas the anti-inflammatory M2 phenotype is minimally represented. The red arrow indicates gene upregulation, whereas the blue arrow shows downregulation. Abbreviations: DAMPs (damage-associated molecular patterns), PAMPs (pathogen-associated molecular patterns), CNS (central nervous system), TNF-α (tumor necrosis factor alpha), iNOS (inducible nitric oxide synthase), IL-1β (interleukin-1 beta), IL-6 (interleukin 6), IL-12 (interleukin 12), IL-23 (interleukin 23), Arg-1 (arginase 1), TGF-β (transforming growth factor beta), and IL-10 (interleukin 10).

Neuroinflammation (Figure 2), as a tightly regulated immunological response within the CNS, comprises a cascade of highly orchestrated molecular events primarily mediated by glial cells as well as other cell types, including endothelial cells, pericytes, infiltrating immune cells, and neurons themselves [1,2,3,4,5]. In the context of chronic neuroinflammation, beyond canonical PRR-mediated activation, cytokine receptor signaling pathways (most notably the JAK/STAT axis) play a key role in orchestrating the transcriptional programs of both pro-inflammatory and anti-inflammatory phenotypes in glial cells [82]. Neuroinflammation begins when PRRs (TLRs, NLRs, and RLRs) detect PAMPs and/or DAMPs in microglial cells (Figure 3) [83,84].

TLR4 binds LPS (from Gram-negative bacteria) or endogenous ligands like HMGB1, S100B proteins, and Hsp proteins [85,86]. Upon ligand binding, TLR4 dimerizes and recruits adaptor proteins (mainly MyD88 and TRIF), thereby initiating two intracellular signaling pathways [87,88]. The MyD88-dependent pathway activates IL-1R-associated kinases (IRAK1 and IRAK4), which in turn phosphorylate TRAF6, culminating in the activation of the IκB kinase (IKK) complex [89]. This phosphorylates the protein IκBα, targeting it for ubiquitination and proteasomal degradation, allowing the dissociation and nuclear translocation of NF-κB dimers (p50/p65) to translocate into the nucleus and initiate transcription of several pro-inflammatory genes such as TNF-α, IL-1β, IL-6, iNOS, and COX-2 [90,91]. In parallel, MAPKs activation triggers the activation of numerous transcription factors, such as AP-1 and CREB, further amplifying the transcriptional response [92,93]. The TRIF-dependent arm of TLR4 signaling activates TBK1 and IKKε kinases, leading to phosphorylation and activation of IRF3, which induces transcription of IFN-γ, essential in antiviral responses and microglial priming [94].

On the other hand, the NLRP3 inflammasome, perhaps the most studied in neuroinflammation, is a tripartite complex formed by the NLRP3 sensor protein, the adaptor ASC, and procaspase-1 [95]. Upon activation by signals such as mitochondrial ROS, K^+^ efflux, and lysosomal destabilization, NLRP3 undergoes conformational change, oligomerizes, and recruits ASC through PYD interactions [96]. Through CARD-CARD interactions, ASC permits the recruitment and activation of procaspase-1, triggering its autocatalytic cleavage into active caspase-1. Thereafter, caspase-1 catalyzes the proteolytic processing of pro-IL-1β and pro-IL-18 into their functional, secreted cytokine forms and contributes to a potent pro-inflammatory environment [97]. Another axis involves purinergic receptors, particularly P2X7 and P2Y12, which respond to extracellular ATP (released during injuries) [98]. P2X7, a ligand-gated cation channel, permits Ca^2+^ and Na^+^ influx and K^+^ efflux upon ATP binding, which serves as a second signal for NLRP3 inflammasome activation [99]. Meanwhile, P2Y12 mediates directed microglial process extension toward injury foci through PI3K/AKT and Rac1 pathways, a mechanism crucial for early neuroimmune surveillance and repair [100].

The arachidonic acid pathway has a profound impact on the neuroinflammatory process. Inflammatory stimuli evoke the expression of cytosolic phospholipase A_2_ (cPLA_2_), which hydrolyzes membrane lipids to release arachidonic acid [101]. This substrate is then converted by COX-2 into prostaglandin H2 (PGH2), which is further metabolized by specific prostaglandin synthases into bioactive prostanoids, mainly prostaglandin E2 (PGE2) [102]. PGE2 binds to EP receptors (EP1-EP4), each coupled to distinct G-proteins, initiating various downstream effects ranging from increased cAMP and Ca^2+^ mobilization to altered gene expression via PKA and CREB [103]. In neurons, PGE2 signaling can exacerbate excitotoxicity by modulating glutamate receptor function, whereas in glial cells, PGE2 promotes cytokine production and impairs the resolution of inflammation [104].

ROS and RNS represent non-cytokine inflammatory mediators with strong effects. Microglial NOX2 becomes activated upon integrin engagement or TLR signaling and catalyzes the one-electron reduction of molecular oxygen to superoxide, which can dismutate into hydrogen peroxide or form peroxynitrite in the presence of NO [105,106]. NO is produced by iNOS, whose transcription is upregulated by NF-κB and STAT1 in response to IFN-γ and TNF-α [107]. Peroxynitrite, a highly reactive oxidant, nitrates tyrosine residues in proteins, damages DNA, and impairs mitochondrial function, thereby promoting neuronal injury and neurodegeneration [108,109].

The complement system constitutes a key component of the innate immune response within the CNS, with classical complement pathway components (especially C1q, C3, and C5a) playing prominent roles in neuroimmune modulation [110]. Under pathological conditions, this pathway becomes aberrantly activated. C1q, the starting molecule of the classical cascade, selectively binds to altered, damaged, or apoptotic synapses and neuronal elements, serving as a molecular tag for downstream complement activation [111]. This engagement triggers the proteolytic cleavage of complement component C3 into its active fragments, C3a and C3b [112]. C3b serves as a potent opsonin, decorating synaptic membranes and apoptotic cells, thus targeting them for phagocytic clearance by microglia via CR3 (also known as CD11b/CD18) [113]. Simultaneously, the cleavage of C5 yields C5a, a pro-inflammatory anaphylatoxin [114]. C3a and C5a exert their biological effects through certain GPCRs (C3aR and C5aR1, respectively) located on glial cells [115,116]. Activation of these receptors enhances microglial chemotaxis, promotes morphological activation, and induces the release of pro-inflammatory cytokines, amplifying the neuroinflammatory milieu [115,116]. While this pathway contributes to host defense and homeostatic clearance under acute conditions, dysregulated activation can lead to highly pathological consequences, including disruption of synaptic integrity and promotion of neuroinflammation [117,118]. Chronic complement activity leads to synaptic pruning, even in the absence of infection and/or injury, thereby compromising synaptic integrity and plasticity [119].

On the other hand, astrocytes, while traditionally viewed as support cells, are active participants in neuroinflammation [18]. Upon stimulation by IL-1β, TNF-α, or TLR agonists, astrocytes upregulate their own expression of pro-inflammatory cytokines, chemokines (such as CCL2 and CXCL10), adhesion molecules (e.g., ICAM-1 and VCAM-1), and acute-phase proteins [68]. These molecules enable leukocyte recruitment across the BBB, whose permeability is compromised during neuroinflammation through the action of MMPs, particularly MMP-2 and MMP-9 [120]. MMPs degrade extracellular matrix components and tight junction proteins like occludin and claudin-5, destabilizing the BBB and enabling infiltration of monocytes, T cells, and neutrophils [121]. Infiltrated immune cells release extra pro-inflammatory mediators, forming a positive feedback loop [122].

Finally, the resolution phase of neuroinflammation is an actively regulated process mediated by SPMs, which are biosynthesized from ω-3 polyunsaturated fatty acids such as docosahexaenoic acid (DHA) and eicosapentaenoic acid (EPA) via the enzymatic activity of lipoxygenases, especially 15-lipoxygenase (15-LOX) [123,124]. These lipid mediators include resolvins (e.g., RvD1 and RvE1), protectins (e.g., PD1), maresins (e.g., MaR1), and lipoxins (LXA4), which exert their pro-resolving effects through binding to certain GPCRs such as GPR32 (RvD1), ALX/FPR2 (LXA4), and ChemR23 (RvE1) [125,126]. The activation of these receptors on glial cells (e.g., microglia) inhibits NF-κB signaling, activates AMPK, and induces PPARγ, thereby promoting a phenotypic shift toward reparative functions [127,128]. These include enhanced phagocytosis of apoptotic cells and myelin debris, suppression of pro-inflammatory cytokines, and increased secretion of neurotrophic factors such as BDNF and IGF-1, facilitating the restoration of homeostasis [129,130,131,132,133]. The failure in SPM biosynthesis or receptor signaling contributes to persistent neuroinflammation, which is a defining pathological feature of some neurodegenerative diseases [134].

## 3. The Dual Role of Neuroinflammation

Neuroinflammation is elicited by a broad spectrum of pathogenic or injurious stimuli, including microbial infections [135], mechanical trauma [136], cerebral ischemia [137], and a range of neurodegenerative processes [138]. This biological phenomenon is characterized by the activation of resident glial cells, which undergo profound phenotypic and functional transformations upon stimulation [7,18]. Activated glial cells release many bioactive molecules that can exert either cytoprotective or cytotoxic effects, depending on the context, intensity, and duration of the inflammatory insult [139,140].

In its acute and transient manifestation, neuroinflammation is widely regarded as a highly coordinated and adaptive physiological response that serves to maintain the structural and functional integrity of the CNS in response to injury or various cellular stressors [141]. This regulated process involves a cascade of molecular and cellular events primarily orchestrated by resident immune cells of the CNS, including microglia and astrocytes, and to a lesser extent by infiltrating peripheral immune cells when the BBB is compromised [142]. One of the principal roles of acute neuroinflammation is the facilitation of phagocytic clearance of apoptotic cells, infectious agents, and cellular debris, thereby preventing the excessive deposition of neurotoxic substances and reducing subsequent damage to the surrounding neural parenchyma [131,143,144,145]. This immune-driven clearance process is further supported by the activation of intracellular signaling cascades that facilitate structural tissue remodeling, thereby facilitating the re-establishment of a permissive environment for neuronal regeneration [146]. Notably, acute neuroinflammation has been shown to stimulate neurogenesis through the upregulation of growth factors such as BDNF and VEGF, which also play a role in promoting angiogenesis, with neurogenesis and vascular remodeling being critical determinants of functional recovery in damaged areas of the CNS [147,148].

Furthermore, this form of neuroinflammatory response plays a key role in the restoration and preservation of CNS homeostasis by acting as a component of the innate immune surveillance system [25]. The CNS has traditionally been considered immunoprivileged, largely due to the protective role of the BBB combined with a limited population of APCs; however, recent studies reveal that an important degree of immunosurveillance is sustained within the CNS, allowing for rapid initiation of responses to both endogenous and exogenous insults [149]. In this context, acute neuroinflammation functions not only as a protective mechanism but also as a dynamic mediator of crosstalk between the neural and immune systems, ensuring rapid detection and mitigation of disturbances to CNS integrity to prevent chronic dysfunction [150]. The resolution phase of this inflammatory response is crucial, marked by the suppression of pro-inflammatory signaling and the induction of anti-inflammatory and pro-resolving pathways, which collectively serve to terminate inflammation and mitigate its potential role in the progression of neurodegenerative pathology [151].

However, when the neuroinflammatory milieu becomes chronic, the same glial-derived mediators that confer protection can instead instigate a cascade of deleterious events [152]. Prolonged activation of microglia and astrocytes promotes a sustained pro-inflammatory milieu, characterized by the persistent upregulation of molecules such as TNF-α, IL-1β, and iNOS [10,11,12]. This pathological state contributes to increased oxidative stress, glutamate-mediated excitotoxicity, and dysregulated synaptic activity [153,154,155]. Chronic neuroinflammation has been implicated in the propagation of some CNS pathologies, including but not limited to AD [156], PD [157], MS [158], and ALS [159]. In these contexts, neuroinflammation is not merely a secondary consequence of neurodegeneration but an active driver of disease progression.

The dichotomous nature of neuroinflammation (simultaneously neuroprotective and neurodestructive) highlights the critical importance of precise spatiotemporal regulation of inflammatory signaling pathways [160]. The molecular crosstalk between neurons, glial cells, endothelial cells, and peripheral immune components ultimately determines the trajectory of the inflammatory response [161]. A pivotal aspect of this process is the tightly regulated interplay between pro-inflammatory (e.g., NF-κB and STAT1) and anti-inflammatory (e.g., IL-10 and TGF-β) signaling cascades, which ultimately dictates whether inflammation is resolved or evolves into a detrimental, chronic state [162].

Emerging evidence also challenges traditional binary classifications of glial phenotypes, such as the M1/M2 dichotomy for microglia, or the A1/A2 paradigm for astrocytes. Instead, reactive glial cells are increasingly recognized as occupying a continuum of activation states, influenced by a confluence of intrinsic and extrinsic factors (including aging, sex, genetic predisposition, systemic immune status, microbiome composition, and environmental exposures) [163,164,165,166,167,168]. The nature of glial phenotypes presents a major obstacle for therapeutic intervention, as broad modulation of inflammatory pathways may unintentionally compromise neuroprotective functions or miss critical functional nuances.

Effective strategies must therefore account for the temporal and spatial heterogeneity of glial responses, as well as the molecular cues driving phenotype transitions. A precise understanding of glial signaling networks is essential to selectively target detrimental processes while preserving or enhancing reparative functions.

## 4. Therapeutic Strategies Targeting Neuroinflammation

Therapeutic strategies targeting neuroinflammation represent a crucial frontier in the management of a wide range of neurological diseases, including AD, PD, and MS, among others [169,170,171]. Due to the multifactorial aspects of neuroinflammation, therapeutic approaches must be tailored to modulate specific molecular and cellular pathways involved in the inflammatory response within the CNS. Current pharmacological interventions can be categorized into established therapies, including nonsteroidal anti-inflammatory drugs (NSAIDs), corticosteroids, and monoclonal antibodies, and into novel experimental therapies, such as microglial modulators and inflammasome inhibitors, each with distinct molecular targets and mechanisms of action.

### 4.1. Established Therapies

#### 4.1.1. Nonsteroidal Anti-Inflammatory Drugs (NSAIDs)

NSAIDs constitute a widely utilized class of pharmacological agents, known for their analgesic, antipyretic, and anti-inflammatory effects [172]. Their main mechanism of action involves the inhibition of COX enzymes, specifically COX-1 and COX-2 [173]. These enzymes are integral to the biosynthetic pathway that converts arachidonic acid (a polyunsaturated fatty acid liberated from membrane phospholipids during cellular stress or injury) into a range of prostanoids, such as prostaglandins, prostacyclins, and thromboxanes [174]. These lipid mediators orchestrate crucial aspects of the inflammatory response, including vasodilation, increased vascular permeability, and the recruitment of immune cells to sites of tissue injury or infection [175]. In contrast with COX-1, which is constitutively expressed and crucial for maintaining physiological functions such as gastrointestinal mucosal protection and renal blood flow, COX-2 is an inducible enzyme whose expression is markedly upregulated by several inflammatory stimuli (e.g., pro-inflammatory cytokines, bacterial pathogens, and injuries) [176]. Within the CNS, COX-2 upregulation occurs in glial cells as well as in neurons under pathological conditions [177,178].

Beyond COX inhibition, many NSAIDs also exert COX-independent anti-inflammatory effects. By interfering with NF-κB activation and nuclear translocation, NSAIDs extend their anti-inflammatory and neuroprotective effects beyond prostaglandin inhibition [179]. Additionally, certain NSAIDs have been shown to modulate peroxisome proliferator-activated receptors (PPARs), particularly PPARγ, providing an additional mechanism through which NSAIDs may attenuate neuroinflammatory processes [180].

Despite their therapeutic potential, the long-term use of NSAIDs is limited by their well-documented adverse effects profile. Long-term NSAID therapy is linked to gastrointestinal complications (including gastritis, peptic ulcer disease, and gastrointestinal bleeding) primarily due to COX-1 inhibition [181]. Renal adverse effects, such as decreased renal perfusion, salt and water retention, and potential acute kidney injury, arise from impaired prostaglandin-mediated renal vasodilation [182]. Moreover, cardiovascular risks, such as hypertension, myocardial infarction, and stroke, are particularly pronounced with selective COX-2 inhibitors (termed as coxibs) [183].

Addressing these safety concerns has prompted the exploration of strategies such as selective COX-2 inhibition [184], dual COX/LOX (lipoxygenase) inhibition [185], development of NO-donating NSAIDs [186], and co-administration of gastroprotective agents like proton pump inhibitors (e.g., omeprazole and lansoprazole) [187]. Nevertheless, achieving an optimal balance between therapeutic efficacy and safety remains a significant challenge in the clinical application of NSAIDs for chronic neuroinflammatory conditions.

Table 1 provides an overview of treatments involving NSAIDs aimed at addressing neuroinflammation in different pathological conditions. This table outlines those NSAIDs used in clinical settings, highlighting their therapeutic effectiveness.

#### 4.1.2. Corticosteroids

Corticosteroids, including dexamethasone and methylprednisolone, exert their anti-inflammatory effects through the activation of the glucocorticoid receptor (GR), a ligand-activated transcription factor that belongs to the nuclear receptor superfamily [197]. Upon entering target cells, corticosteroids bind to the GR in the cytoplasm, inducing its conformational change and dissociation from Hsp proteins (e.g., HSP70 and HSP90), facilitating its translocation into the nucleus [198]. Once in the nucleus, the GR–ligand complex binds to specific DNA sequences known as glucocorticoid response elements (GREs) located in the promoter regions of some target genes, recruiting coactivators such as steroid receptor coactivator-1 (SRC-1) and p300 [199,200,201]. This interaction promotes the transcription of anti-inflammatory genes (e.g., IL-10) while inhibiting the expression of pro-inflammatory cytokines such as IL-1β, TNF-α, and IL-6 [202,203]. Furthermore, the GR exerts transcriptional interference with several transcription factors, especially NF-κB (by binding to the p65 subunit of NF-κB, it prevents its translocation to the nucleus and DNA binding) [204] and AP-1 (by sequestering AP-1 activators-such as c-Jun-, inhibiting their ability to promote the transcription of pro-inflammatory genes) [205].

Additionally, corticosteroids inhibit the expression of some adhesion molecules, such as ICAM-1 and VCAM-1, thereby reducing immune cell recruitment to inflamed tissues [206,207]. Corticosteroids also promote the apoptosis of activated immune cells, particularly T and B lymphocytes, through the induction of pro-apoptotic proteins including BIM and FASL, eliciting some extrinsic and intrinsic apoptotic pathways [208,209]. In contrast, corticosteroids decrease the permeability of the BBB, preventing the infiltration of peripheral immune cells into the CNS, thereby limiting neuroinflammation [210].

Despite their anti-inflammatory actions, corticosteroids are associated with numerous adverse effects, including immunosuppression [211], metabolic alterations [212], osteoporosis [213], and neuropsychiatric disturbances [214], especially with prolonged use, underscoring the importance of vigilant monitoring and dose modulation in clinical settings.

Table 2 presents an overview of corticosteroid treatments targeting neuroinflammation in different pathological conditions. It highlights the corticosteroids utilized in clinical settings, emphasizing their therapeutic effectiveness.

### 4.2. Experimental Approaches

#### 4.2.1. Monoclonal Antibodies

Monoclonal antibody (mAb) therapies have emerged as a groundbreaking strategy in the treatment of neuroinflammatory disorders, offering unparalleled specificity in molecular targeting, thereby enabling precise modulation of pathological pathways within the CNS [222]. A leading example of this therapeutic progress is natalizumab, an anti-α4 integrin mAb. α4 integrin is a cell adhesion molecule expressed on the surface of leukocytes, including T-cells, monocytes, and granulocytes [223]. By binding to α4 integrin, natalizumab blocks its interaction with VCAM-1, thereby preventing the adhesion and subsequent transmigration of leukocytes across the BBB [224]. This mechanism is particularly significant in the context of MS, where autoreactive immune cells infiltrate the CNS, triggering inflammation that leads to demyelination and neurodegeneration [225].

Similarly, monoclonal antibodies targeting TNF-α have demonstrated therapeutic efficacy in neuroinflammatory diseases. Anti-TNF-α antibodies, including infliximab and adalimumab, neutralize both soluble and membrane-bound forms of TNF-α, thereby inhibiting its downstream signaling pathways [226,227]. These antibodies prevent the activation of TNF receptors (TNFR1 and TNFR2), which are involved in the induction of inflammatory cascades, oxidative stress, and neuronal cell death [228,229]. Its inhibition can impair the immune response, leaving patients susceptible to opportunistic infections, such as tuberculosis, fungal infections, and viral reactivation [230,231,232].

In recent years, mAbs targeting other cytokines implicated in chronic CNS inflammation have gained attention. Canakinumab, a mAb that selectively targets and neutralizes IL-1β, has shown promise in modulating the IL-1β signaling pathway. Through the inhibition of IL-1β binding to its receptor (IL-1R), canakinumab reduces the downstream production of other inflammatory mediators, including IL-6, IL-8, and MMPs, which contribute to BBB disruption and neuronal damage [233,234,235]. Similarly, tocilizumab, a mAb that antagonizes the IL-6 receptor (IL-6R), has been developed as a treatment for autoimmune diseases, and its potential in modulating neuroinflammation is under investigation [236]. Blocking IL-6R signaling reduces the expression of pro-inflammatory cytokines in the brain, thus providing a targeted approach to attenuate neuroinflammation [237].

Another area of significant interest is the development of monoclonal antibodies targeting Aβ plaques, which are critical in the pathogenesis of AD. Aβ plaques are neurotoxic protein aggregates that accumulate in the brains of individuals with AD, driving neuroinflammation, synaptic dysfunction, and neurodegeneration [238]. The accumulation of Aβ plaques triggers the activation of glial cells, leading to the release of many pro-inflammatory cytokines and the exacerbation of neuronal injury [239]. mAbs, such as aducanumab, have been developed to specifically target and promote the clearance of Aβ plaques [240]. These antibodies recognize both soluble and insoluble forms of Aβ, facilitating their clearance via microglial phagocytosis or by enhancing amyloid elimination [241]. By reducing Aβ accumulation and mitigating the associated neuroinflammatory response, aducanumab has shown promise in slowing disease progression and improving cognitive function in AD patients [242].

Moreover, additional mAbs have been introduced to target other key inflammatory mediators in neuroinflammatory disorders. Eculizumab, a mAb targeting the complement protein C5, has been shown to attenuate the activation of the complement system, which plays a role in the pathogenesis of diseases such as neuromyelitis optica spectrum disorder (NMOSD) [243]. Through the inhibition of C5, eculizumab prevents the assembly of the membrane attack complex (MAC), thereby diminishing the inflammation and tissue damage associated with complement activation [244]. Further advances have been made in targeting the IL-17 pathway, a key driver of autoimmune neuroinflammation. Monoclonal antibodies such as secukinumab and ixekizumab, which target IL-17A, have been successfully used in the treatment of psoriasis and ankylosing spondylitis [245,246], and their potential in CNS disorders is under exploration [247,248,249]. Finally, mAbs targeting IL-23, a cytokine upstream of IL-17 production, have shown promise in reducing inflammation in preclinical models of neuroinflammatory conditions [250].

The development of mAb therapies for neuroinflammatory disorders is continuously advancing, with ongoing research aimed at discovering new targets and optimizing therapeutic efficacy. Strategies include the refinement of antibody selectivity, improving delivery across the BBB, and minimizing adverse effects, such as increased susceptibility to infections [251]. Furthermore, combination therapies targeting numerous pro-inflammatory pathways are under investigation as potential strategies to improve clinical outcomes in patients with complex neuroinflammatory disorders [252]. As our understanding of the molecular mechanisms underlying neuroinflammation continues to deepen, mAb-based therapies hold great promise in the treatment of a broad spectrum of CNS disorders.

Table 3 details the treatments previously referenced, assessed in certain clinical trials, alongside additional interventions not previously cited, to highlight the broad spectrum of therapeutic strategies available for neuroinflammation-associated disorders.

#### 4.2.2. Other Innovative Therapeutic Strategies

Recent advances in the development of novel microglial modulators offer promising therapeutic strategies designed to restore microglial function and completely mitigate the harmful consequences of dysregulated activation. One such class of modulators includes colony-stimulating factor 1 receptor (CSF1R) inhibitors, such as PLX5622. CSF1R is a receptor critical for microglial survival, proliferation, and maintenance, as it transduces signals required for the development of microglia in the CNS [268]. Inhibition of CSF1R signaling by these compounds results in the depletion or phenotypic reprogramming of microglia, favoring their transition to a quiescent or neuroprotective state [269]. This reprogramming reduces the production of pro-inflammatory cytokines and ROS, thus mitigating the neurotoxic effects of overactive microglia [270,271]. CSF1R inhibition has been shown to impair the survival signals necessary for microglial proliferation, effectively reducing microglial numbers and dampening chronic neuroinflammation [272].

Another promising approach to modulate microglial activity involves targeting the fractalkine receptor (CX3CR1), which mediates communication between neurons and microglia. CX3CR1 antagonists are designed to restore homeostatic interactions between microglia and neurons, thereby mitigating the adverse consequences of excessive microglial activation, including aberrant synaptic pruning and neurotoxicity [273,274]. This strategy seeks to enhance the neuroprotective functions of microglia while preventing them from overreacting to minor stimuli, a characteristic factor in neuroinflammatory diseases [275]. Moreover, pharmacological agents that enhance PPARγ signaling, like pioglitazone, have shown promise in modulating microglial polarization [276]. On the other hand, selective NLRP3 inhibitors, like MCC950, block the activation of NLRP3 by preventing its ATPase-dependent oligomerization. This inhibition attenuates caspase-1 activation and the subsequent release of pro-inflammatory cytokines without interfering with other aspects of the innate immune response [277]. In preclinical models, NLRP3 inhibition has been shown to reduce microglial activation, BBB disruption, and neuronal death, highlighting its potential as a therapeutic approach in neuroinflammatory disorders [278].

Beyond direct inflammasome inhibition, other experimental strategies focus on targeting downstream effectors of inflammasome activation, such as gasdermin D. Gasdermin D is the executor of pyroptosis, a form of programmed cell death that is often associated with inflammatory responses [279]. Pyroptosis exacerbates tissue injury and contributes to disease progression in neuroinflammatory conditions. By inhibiting gasdermin D-mediated pyroptosis, these strategies aim to minimize neuronal damage and preserve tissue integrity [280].

Collectively, these innovative strategies constitute a multifactorial approach to modulating neuroinflammation, preserving neural tissue integrity, and enhancing therapeutic outcomes in neurodegenerative disorders. By selectively reprogramming microglia and inhibiting harmful inflammatory processes, these therapies hold great potential for mitigating the devastating effects of chronic neuroinflammation and preserving CNS function.

## 5. Future Directions and Research Gaps

Although considerable progress has been made in elucidating the molecular pathways and cellular mechanisms involved in neuroinflammation, several critical challenges persist that limit the advancement of effective diagnostic tools and therapeutic interventions. Among the foremost research imperatives is the identification and rigorous validation of highly specific, sensitive, and reliable biomarkers capable of distinguishing homeostatic and beneficial neuroinflammatory activity from chronic and huge neuroinflammatory responses [281]. Current clinical and experimental methodologies lack the resolution to differentiate adaptive immune activation (crucial for tissue repair and neuroprotection) from maladaptive inflammatory cascades that exacerbate neuronal injury and promote neurodegeneration [282]. The development of such biomarkers would enable earlier and precise detection of neuroinflammatory components in neurological pathologies, support the stratification of patients in clinical trials, and permit real-time monitoring of therapeutic efficacy [283].

Another major limitation in the field is the lack of physiologically relevant and translationally predictive experimental models. Conventional in vitro models largely fail to replicate the multicellular interactions, spatial organization, and dynamic microenvironment characteristic of the human CNS [284]. Likewise, widely used animal models often lack construct or face validity, especially in the context of chronic neurodegenerative diseases, aging, and comorbid systemic conditions [285]. A growing body of evidence underscores the urgent need for more sophisticated models (such as human-derived organoids and microfluidic brain-on-a-chip platforms), and genetically modified animal models that more faithfully emulate the human neuroimmune microenvironment (to effectively bridge the translational divide between preclinical and clinical research) [286,287]. These models should incorporate innate and adaptive immune responses, temporal dynamics, and the influence of systemic immune modulation to provide a more comprehensive understanding of neuroinflammatory processes across different disease stages.

Moreover, the long-term consequences of modulating neuroinflammation (especially via broad-spectrum or prolonged anti-inflammatory interventions) remain insufficiently characterized. While suppression of inflammation may yield short-term symptomatic relief or neuroprotection, accumulating evidence suggests that persistent inhibition of immune signaling within the CNS might interfere with critical physiological functions, such as synaptic plasticity, neurogenesis, and clearance of cellular debris [288,289,290]. Glial cells exhibit a high degree of functional plasticity, and their roles in disease are highly context-dependent. Therefore, indiscriminate targeting of these glial populations or their associated cytokine networks may lead to unintended consequences, including impaired neural repair, increased susceptibility to infections, or disruption of neuroimmune homeostasis [288,291]. Additionally, the long-term impact of modulating neuroinflammation, through the application of broad-spectrum or sustained anti-inflammatory treatments, remains inadequately defined [292].

Furthermore, clinical studies are crucial to evaluate the safety, efficacy, and mechanistic impact of anti-inflammatory and immunomodulatory therapies over extended time periods [293]. This research should incorporate a multimodal approach, combining neuroimaging, fluid biomarkers, electrophysiology, and comprehensive neurocognitive assessments, to accurately capture the multifaceted effects of treatment. Integrating systems biology, single-cell transcriptomics, and machine learning may also offer novel insights into patient-specific inflammatory signatures and therapeutic responsiveness, paving the way toward personalized medicine in the treatment of neuroinflammatory disorders [294,295,296].

In summary, addressing these research gaps requires a multidisciplinary effort that spans basic neuroscience, immunology, systems biology, and clinical research. The future of neuroinflammation research depends on the development of innovative tools to elucidate its dualistic nature, the creation of precise and representative models, and a comprehensive understanding of how therapeutic modulation influences the delicate balance between neuroprotection and neurotoxicity throughout the lifespan and within the context of complex neurological disorders.

## 6. Conclusions

Neuroinflammation is a complex and multifaceted process that plays both protective and deleterious roles in the CNS. This dual nature underscores its critical involvement in the pathogenesis and progression of some neurological disorders, including neurodegenerative diseases, TBI, and autoimmune conditions. Mechanistically, neuroinflammation is orchestrated by a dynamic interplay of glial cell activation, cytokine release, and BBB dysfunction, which contribute to neuronal damage or repair depending on the context.

While acute neuroinflammatory responses might serve protective and regenerative functions, chronic or dysregulated inflammation is strongly associated with neuronal degeneration. Therefore, therapeutic strategies targeting neuroinflammation must be calibrated to preserve its beneficial aspects while mitigating its harmful consequences.

Emerging interventions, like immunomodulatory agents, cytokine inhibitors, and glial-targeted therapies, exhibit encouraging results in preclinical and clinical studies. A deeper understanding of the molecular pathways governing neuroinflammation will be pivotal for developing precision-based treatments tailored to individual disease states and stages.

## Figures and Tables

**Table 1:** NSAID-based therapeutic approaches for pathologies associated with neuroinflammatory processes. Abbreviations: MS (multiple sclerosis), AD (Alzheimer’s disease), PD (Parkinson’s disease), RR (relative risk), CI (confidence interval), OR (odd ratio), NSAID (non-steroidal anti-inflammatory drug), TLR4 (Toll-Like receptor 4), TNF-α (tumor necrosis factor alpha), BDNF (brain-derived neurotrophic factor), Nrf-2 (nuclear factor erythroid 2-related factor 2), UPDRS (Unified Parkinson’s Disease Rating Scale), TBI (traumatic brain injury), COX (cyclooxygenase), ALS (amyotrophic lateral sclerosis), TDP-43 (TAR DNA-binding protein 43), and LC3 (microtubule-associated protein 1A/1B-light chain 3).

Disease	NSAID Employed	Effects	References
Multiple Sclerosis (MS)	Aspirin	Aspirin exhibits several potential therapeutic benefits, including anti-inflammatory effects, promotion of oligodendrocyte precursor cell proliferation, and improvement of symptoms such as fatigue. Nevertheless, its use requires caution due to potential adverse effects, notably an increased risk of hemorrhagic stroke and inhibition of mitochondrial complex I activity, both of which are particularly relevant in this pathology.	[188]
Alzheimer’s Disease (AD)	Diclofenac	Diclofenac use was found to be associated with a reduced incidence of AD and slower progression of cognitive decline, underscoring the necessity for further research into the therapeutic potential of diclofenac in AD.	[189]
	Aspirin	The findings of this analysis suggest a genetically mediated protective effect of aspirin use against AD, potentially modulated by coronary heart disease, blood pressure, and lipid levels.	[190]
Parkinson’s Disease (PD)	Ibuprofen	Over six years of follow-up, 291 incident PD cases were identified. Ibuprofen use was associated with a significantly lower PD risk (RR = 0.62; 95% CI 0.42–0.93; *p* = 0.02), with a dose–response relationship (*p* = 0.01). In another study, the ORs for PD occurrence in patients who took NSAIDs, ibuprofen, and non-aspirin NSAIDs were 0.88 [95% CI (0.80–0.97), *p* = 0.01], 0.73 [95% CI (0.53–1.00), *p* = 0.05], and 0.85 [95% CI (0.7–0.97), *p* = 0.01], respectively.	[191,192]
	Celecoxib	The celecoxib group exhibited a significant reduction in the levels of TLR4 (*p* = 0.004), TNF-α (*p* = 0.042), and α-Syn (*p* = 0.004), as well as a notable increase in the levels of BDNF (*p* = 0.0005) and Nrf-2 (*p* = 0.004), as compared to the control group. Additionally, UPDRS scores were significantly lower in the celecoxib group (*p* < 0.05).	[193]
Traumatic Brain Injury (TBI)	Celecoxib	Celecoxib use in patients with TBI was found to be associated with a significantly lower 1-year mortality rate (6.4% vs. 10.0%; OR 0.61; 95% CI 0.46–0.80; *p* = 0.0003) and higher survival rate (96.1% vs. 93.1%; *p* < 0.0001) compared to the control group. Celecoxib was also linked to reduced rates of gastrostomy tube dependence, seizure activity, and myocardial infarction.	[194]
Amyotrophic Lateral Sclerosis (ALS)	Aspirin	Multivariate analysis revealed that aspirin use was independently inversely associated with ALS risk after adjustment for diphenhydramine, mefenamic acid, and steroid use. This inverse association was predominantly observed in individuals over 55 years of age.	[195]
	Celecoxib (plus ciprofloxacin)	Biomarker analyses revealed significant PrimeC (celecoxib combined with ciprofloxacin)-induced alterations in neuron-derived exosomal TDP-43 and LC3 levels, the latter being a key marker of autophagy. These results support the safety and tolerability of PrimeC in ALS and offer preliminary evidence of its biological activity.	[196]

**Table 2:** Corticosteroid-based therapeutic strategies for disorders linked to neuroinflammatory processes. Abbreviations: MS (multiple sclerosis), RRMS (relapsing–remitting multiple sclerosis), MRI (magnetic resonance imaging), and IFNβ-1a (interferon beta-1a).

Disease	Corticosteroid Employed	Effects	References
Multiple Sclerosis (MS)	Prednisone	Durelli et al. [215] conducted a study involving 23 patients with RRMS, who were treated over a 15-day period with either a placebo or methylprednisolone, initiated at a dose of 15 mg/kg/day and gradually tapered. Following this initial phase, all patients received oral prednisone at a dose of 100 mg/day, tapered over 120 days. The authors reported a statistically significant clinical benefit (*p* < 0.05) in favor of methylprednisolone treatment on days 5, 10, and 15.	[215]
	Prednisolone	Methylprednisolone therapy in MS markedly reduces both the severity and duration of clinical relapses and the number of gadolinium-enhancing T1 lesions on MRI. In a cohort of 13 MS patients undergoing 31 treatment cycles over a mean of 50 weeks, 609 active lesions were identified across 195 MRI scans. Post-treatment imaging revealed a 78% immediate reduction in enhancing lesions, indicating transient restoration of blood–brain barrier integrity and inflammatory suppression. Re-enhancement of lesions was rare. The therapeutic effect persisted for approximately 10 weeks.	[216]
	Methylprednisolone	In 2009, a randomized study was published involving 181 patients with RRMS, all of whom were receiving treatment with IFNβ-1a. Participants were assigned to receive one of three adjunctive treatments over a two-year period: (1) placebo, (2) oral azathioprine (50 mg/day), or (3) oral azathioprine (50 mg/day) combined with oral prednisolone (10 mg every other day). The combination of prednisolone and azathioprine was associated with a 20% reduction in the annualized relapse rate.	[217]
Autoimmune Encephalitis	Prednisone	Employed as a first-line therapy, a common approach involves initiating oral prednisone at a dose of 1–2 mg/kg/day immediately following the completion of acute treatment, followed by a gradual taper over several weeks to months.	[218]
	Methylprednisolone	As a first-line therapy for autoimmune encephalitis, treatment typically consists of intravenous methylprednisolone at 1 g per day for 5 consecutive days, followed by oral prednisone at 1 mg/kg/day (up to a maximum of 60–80 mg/day), with a gradual tapering protocol extending over 3 to 6 months.	[219]
		Employed as a first-line treatment in infantile autoimmune encephalitis, intravenous methylprednisolone is administered at a dose of 30 mg/kg/day for 3 to 5 days.	[220]
	Dexamethasone (plus immunoglobulins)	Therapy with dexamethasone and intravenous immunoglobulin resulted in significant clinical improvement during both acute and non-acute phases of the disease. At the final follow-up, 90.2% of patients exhibited a favorable clinical outcome, with 43.9% achieving full recovery and a relapse rate of only 2.4%. No severe adverse events were observed.	[221]

**Table 3:** Experimental immunotherapeutic treatments targeting various CNS disorders characterized by neuroinflammation. Abbreviations: mAb (monoclonal antibody), MS (multiple sclerosis), CD20 (cluster of differentiation 20), CD52 (cluster of differentiation 52), RRMS (relapsing–remitting multiple sclerosis), PPMS (primary progressive multiple sclerosis), NMOSD (neuromyelitis optica spectrum disorder), C5 (complement component 5), IL-6R (interleukin 6 receptor), CD19 (cluster of differentiation 19), AD (Alzheimer’s disease), PD (Parkinson’s disease), CNS (central nervous system), NPSLE (neuropsychiatric systemic lupus erythematosus), AAV (anti-neutrophil cytoplasmic antibody-associated vasculitis), and BAFF (B-cell activating factor).

Disease	mAb Employed	Clinical Trial Phase	Effects	References
Multiple Sclerosis (MS)	Natalizumab (Anti-α4 integrin)	Phase III	Inhibited leukocyte migration Reduced relapses	[253]
	Ocrelizumab (Anti-CD20)	Phase III	Caused B-cell depletion Effective in RRMS and PPMS	[254]
	Alemtuzumab (Anti-CD52)	Phase III	Produced long-lasting lymphocyte depletion	[255]
	Ofatumumab (Anti-CD20)	Phase III	Effective in reducing disease activity	[256]
Neuromyelitis Optica Spectrum Disorder (NMOSD)	Eculizumab (Anti-C5)	Phase III	Inhibited the complement cascade Prevented attacks	[257]
	Satralizumab (Anti-IL-6R)	Phase III	Reduced relapse risk	[258]
	Inebilizumab (Anti-CD19)	Phase II/III	Reduced relapse rate	[259]
Autoimmune Encephalitis	Rituximab (Anti-CD20)	Phase II	Induced B-cell depletion Improved psychiatric and neurologic symptoms	[260]
	Tocilizumab (Anti-IL-6R)	Phase II	Effective for refractory or relapsing cases	[261]
Alzheimer’s Disease (AD)	Aducanumab (Anti-Aβ plaques)	Phase III/Approved	Reduced Aβ plaques	[240]
	Lecanemab (Anti-Aβ plaques)	Phase III	Provided modest cognitive benefit	[262]
	Donanemab (Anti-Aβ plaques)	Phase III	Slowed progression in early AD	[263]
Parkinson’s Disease (PD)	Prasinezumab (Anti-α-synuclein)	Phase II	Targeted misfolded protein aggregates	[264]
	Cinpanemab (Anti-α-synuclein)	Phase II	Under investigation	[265]
CNS Vasculitis	Rituximab (Anti-CD20)	Phase II	Served as an alternative to cyclophosphamide in AAV-related cases	[266]
Neuropsychiatric Systemic Lupus Erythematosus (NPSLE)	Belimumab (Anti-BAFF)	Phase III	Caused B-cell suppression Reduced neuropsychiatric flare frequency	[267]

## Data Availability

Not applicable.

## Abbreviations

The following abbreviations are used in this manuscript:

15-LOX	15-lipoxygenase
A1	A1 phenotype astroglia
A2	A2 phenotype astroglia
AAV	Anti-neutrophil cytoplasmic antibody-associated vasculitis
AD	Alzheimer’s disease
ALX/FPR2	ALX/formyl peptide receptor 2
AMPK	AMP-activated protein kinase
AP-1	Activator protein 1
Arg-1	Arginase 1
ASC	Apoptosis-associated speck-like protein containing a CARD
APC	Antigen-presenting cell
ATP	Adenosine triphosphate
Aβ	Amyloid beta
BAFF	B-cell activating factor
BBB	Blood–brain barrier
BDNF	Brain-derived neurotrophic factor
BIM	Bcl-2 interacting mediator of cell death
C1q	Complement component 1q
C3	Complement component 3
C3a	Complement component 3a
C3aR	Complement component 3a receptor
C3b	Complement component 3b
C5	Complement component 5
C5a	Complement component 5a
C5aR1	Complement component 5a receptor 1
cAMP	Cyclic adenosine monophosphate
CARD	Caspase activation and recruitment domain
CCL2	Chemokine (C-C motif) ligand 2
CD11b	Cluster of differentiation 11b
CD18	Cluster of differentiation 18
CD19	Cluster of differentiation 19
CD20	Cluster of differentiation 20
CD52	Cluster of differentiation 52
CD200	Cluster of differentiation 200
ChemR23	Chemokine-like receptor 1
CI	Confidence interval
c-Jun	c-Jun proto-oncogene
CNS	Central nervous system
COX	Cyclooxygenase
COX-1	Cyclooxygenase 1
COX-2	Cyclooxygenase 2
cPLA2	Cytosolic phospholipase A2
CR3	Complement receptor 3
CREB	cAMP response element-binding protein
CSF1R	Colony-stimulating factor 1 receptor
CX3CL1	C-X3-C motif chemokine ligand 1
CX3CR1	C-X3-C motif chemokine receptor 1
CXCL10	C-X-C motif chemokine ligand 10
DAMP	Damage-associated molecular pattern
DHA	Docosahexaenoic acid
DNA	Deoxyribonucleic acid
EP	E-prostanoid receptor (PGE2 receptor)
EP1	E-type prostanoid receptor 1
EP2	E-type prostanoid receptor 2
EP3	E-type prostanoid receptor 3
EP4	E-type prostanoid receptor 4
EPA	Eicosapentaenoic acid
ERK	Extracellular signal-regulated kinase
FASL	Fas ligand
GPCR	G protein-coupled receptor
GPR32	G protein-coupled receptor 32
GR	Glucocorticoid receptor
GRE	Glucocorticoid response element
HMGB1	High mobility group box 1
Hsp	Heat shock protein
HSP70	Heat shock protein 70
HSP90	Heat shock protein 90
ICAM-1	Intercellular adhesion molecule 1
IFN-β	Interferon beta
IFNβ-1a	Interferon beta-1a
IGF-1	Insulin-like growth factor 1
IKK	IκB kinase
IKKε	IκB kinase epsilon
IL-10	Interleukin 10
IL-12	Interleukin 12
IL-17	Interleukin 17
IL-17A	Interleukin 17A
IL-1R	Interleukin 1 receptor
IL-1α	Interleukin 1 alpha
IL-1β	Interleukin 1 beta
IL-23	Interleukin 23
IL-6	Interleukin 6
IL-6R	Interleukin 6 receptor
iNOS	Inducible nitric oxide synthase
IRAK1	Interleukin 1 receptor-associated kinase 1
IRAK4	Interleukin 1 receptor-associated kinase 4
IRF	Interferon regulatory factor
IRF3	Interferon regulatory factor 3
IκBα	Inhibitor of κB alpha
JAK	Janus kinase
JNK	c-Jun N-terminal kinase
LC3	Microtubule-associated protein 1A/1B-light chain 3
LD	Lafora disease
LOX	Lipoxygenase
LXA4	Lipoxin A4
M1	M1 phenotype microglia
M2	M2 phenotype microglia
mAb	Monoclonal antibody
MaR1	Maresin 1
MAC	Membrane attack complex
MAPK	Mitogen-activated protein kinase
MMP	Matrix metalloproteinase
MMP-2	Matrix metalloproteinase 2
MMP-9	Matrix metalloproteinase 9
MRI	Magnetic resonance imaging
MS	Multiple sclerosis
MyD88	Myeloid differentiation primary response 88
NCLs	Neuronal ceroid lipofuscinoses
NF-κB	Nuclear factor kappa-light-chain-enhancer of activated B-cells
NLR	NOD-like receptor
NLRP3	NLR family pyrin domain containing 3
NMDA	N-methyl-D-aspartate
NMOSD	Neuromyelitis optica spectrum disorder
NO	Nitric oxide
NOX2	NADPH oxidase 2
NPSLE	Neuropsychiatric systemic lupus erythematosus
Nrf-2	Nuclear factor erythroid 2-related factor 2
NSAID	Non-steroidal anti-inflammatory drug
OPC	Oligodendrocyte precursor cell
OR	Odds ratio
P2X7	Purinergic receptor P2X, ligand-gated ion channel 7
P2Y12	Purinergic receptor P2Y, G-protein coupled, 12
p300	E1A binding protein p300
p38	p38 mitogen-activated protein kinase
p50	NF-κB p50 subunit
p53	Tumor protein 53
p65	NF-κB p65 (RelA) subunit
PAMP	Pathogen-associated molecular pattern
PD	Parkinson’s disease
PD1	Protectin D1
PGE2	Prostaglandin E2
PGH2	Prostaglandin H2
PI3K/AKT	Phosphoinositide 3-kinase/Protein kinase B pathway
PPAR	Peroxisome proliferator-activated receptor
PPARγ	Peroxisome proliferator-activated receptor gamma
pro-IL-18	Pro-interleukin 18
pro-IL-1β	Pro-interleukin 1 beta
PRR	Pattern recognition receptor
PYD	Pyrin domain
Rac1	Ras-related C3 botulinum toxin substrate 1
RLR	RIG-I-like receptor
RNS	Reactive nitrogen species
ROS	Reactive oxygen species
RR	Relative risk
RRMS	Relapsing–remitting multiple sclerosis
RvD1	Resolvin D1
RvE1	Resolvin E1
S100B	S100 calcium-binding protein B
SPM	Specialized pro-resolving mediators
SRC-1	Steroid receptor coactivator-1
STAT	Signal transducer and activator of transcription
STAT1	Signal transducer and activator of transcription 1
TBI	Traumatic brain injury
TBK1	TANK-binding kinase 1
TDP-43	TAR DNA-binding protein 43
TGF-β	Transforming growth factor beta
TLR	Toll-like receptor
TLR4	Toll-like receptor 4
TNFR1	Tumor necrosis factor receptor 1
TNFR2	Tumor necrosis factor receptor 2
TNF-α	Tumor necrosis factor alpha
TRAF6	TNF receptor-associated factor 6
TRIF	TIR-domain-containing adapter-inducing interferon beta
UPDRS	Unified Parkinson’s Disease Rating Scale
VCAM-1	Vascular cell adhesion molecule 1
VEGF	Vascular endothelial growth factor
